# Transcription-dependent polyadenylation signal activity contributes to transcriptomic regulation during Forskolin-induced BeWo cell syncytialization

**DOI:** 10.3389/fcell.2026.1958080

**Published:** 2026-09-25

**Authors:** Juan Liu, Guolin Ye, Xin Liu, Haiming Jing, Shan Gao, Zhongyuan Deng, Junyu Ning

**Affiliations:** 1 Beijing Key Laboratory of Diagnostic and Traceability Technologies for Food Poisoning, Beijing Center for Disease Prevention and Control, Beijing, China; 2 School of Public Health, Capital Medical University, Beijing, China; 3 School of Agriculture and Biomanufacturing, Zhengzhou University, Zhengzhou, China; 4 Biology and Agriculture Research Center, University of Science and Technology Beijing, Beijing, China

**Keywords:** polyadenylation, polyadenylation signals, transcriptional activity, placenta, trophoblast syncytialization

## Abstract

Polyadenylation, a core transcriptional step governed by polyadenylation signals (PAS), functionally interacts with transcription. Nevertheless, how PAS sequences respond to variable transcriptional output remains poorly characterized. Here, we precisely modulated cellular transcription levels by integrating SV40 promoter truncation with a Tet-On dual-luciferase reporter system. Diminished polyadenylation efficiency accompanies lowered promoter activity, and graded transcription assays revealed a tiered PAS processing model: canonical and suboptimal PASs maintain stable 3′-end processing at high transcription levels, whereas non-canonical PASs display compromised processing efficiency with rising transcription. During forskolin(FSK)-induced BeWo cell syncytialization, endogenous trophoblast fusion genes carrying potent canonical PASs were selectively mobilized upon transcriptional activation to enrich differentiation transcripts and sustain orderly syncytiotrophoblast maturation. Together, these findings reveal a hierarchical transcription-polyadenylation axis that associate with placental trophoblast syncytialization. They fill key gaps in mRNA processing and provide a foundation for studying the transcriptional regulatory mechanisms underlying trophoblast differentiation.

## Introduction

Messenger RNA (mRNA) biogenesis in eukaryotic cells relies on a tightly coordinated, multistep cascade consisting of transcription initiation, elongation, and termination, followed by essential co-transcriptional RNA maturation reactions, including 5′capping, precursor mRNA splicing and 3'-terminal poly(A) tails synthesis. All these sequential biochemical reactions are functionally coupled and governed by layered regulatory networks, ensuring the high-fidelity and efficient transmission of genetic information (Nag et al., 2006). As an obligatory maturation step for nearly all protein-coding transcripts, mRNA polyadenylation broadly modulates multiple stages of gene expression. This process is based on two very tightly interconnected biochemical reactions. First, nascent pre-mRNA undergoes site-specific cleavage at conserved 3′processing motif to generate a free 3′hydroxyl terminus. Second, poly(A) polymerase (PAP) catalyzes template-independent addition of adenosine monophosphate (AMP) residues to this free terminus, ultimately forming the poly(A) tail (Rehfeld et al., 2013).

Polyadenylation serves two core and evolutionarily conserved functions: defining the 3′boundary of mature mRNA and facilitating transcriptional termination. Upon recognition of polyadenylation signal sequence (PAS), RNA polymerase II (Pol II) undergoes transcriptional pausing. This transient pause creates a permissive time window for the recruitment and assembly of the complete 3'-end processing machinery, which subsequently cleaves nascent RNA chain. RNA cleavage further triggers the dissociation of Pol II from DNA templates and full termination of transcription. Beyond transcriptional regulation, polyadenylation sets a functional link with mRNA nuclear export pathways. The poly(A) tail acts as a docking platform for sequence-specific RNA binding proteins, which promotes assembly of export-competent messenger ribonucleoprotein (mRNP) complexs. Mature mRNPs are subsequently recognized by nuclear pore complex subunits and efficiently transported from the nuclear compartment to the cytoplasmic space (Rehfeld et al., 2013). By contrast, transcripts lacking 3'poly(A) extensions fail to form transport-competent mRNPs and are thus retained within the nuclear compartment, preventing their cytoplasmic translocation (Miao and Hu, 2025). Accordingly, poly(A) tail addition is not merely a mandatory pre-mRNA processing step, but a central regulatory module that remodels global gene expression landscapes in eukaryotes.

Polyadenylation accuracy and efficiency are precisely controlled by the interplay between *cis*-regulatory sequences and *trans*-acting protein factors. As the primary *cis*-element and a master checkpoint for gene expression, the PAS mediates the recruitment of processing enzymes and auxiliary regulators into catalytically active ribonucleoprotein assemblies (Lin et al., 2021). Approximately 70% of human genes transcripts carry canonical PAS hexamers (AAUAAA/AUUAAA), whereas the remaining transcripts depend on non-canonical alternative PAS architectures or PAS-independent regulatory mechanisms to accomplish 3'-end processing (Di Giammartino et al., 2011). Efficient pre-mRNA cleavage and poly(A) synthesis require intact functional PAS motifs as well as coordinated assembly of multisubunit protein complexes. The core catalytic machinery comprises cleavage and polyadenylation specificity factor (CPSF), cleavage stimulation factor (CstF), and cleavage factor Im (CFIm). Meanwhile, auxiliary regulators including splicing cofactors, transcriptional modulators, and chromatin remodelers further enrich the hierarchical complexity of this regulatory network (Tang and Zhou, 2022). Sun et al. demonstrated that the canonical AAUAAA motif, typically located 10–30 nucleotides upstream of the cleavage site, directly recruits CPSF to initiate pre-mRNA cleavage and poly(A) tail addition. Conversely, divergent non-canonical PAS motifs (e.g., AUUAAA, UAUAAA) reduce the substrate affinity of CPSF and thereby attenuate overall polyadenylation efficiency (Sun et al., 2017). In addition to *cis*-sequence signals, *trans*-acting RNA-binding proteins, particularly poly(A) polymerases (PAPs) and nuclear poly(A)-binding protein II (PABPII), are equally critical for robust poly(A) synthesis. CPSF and PABPII cooperatively potentiate PAP catalytic activity to sustain processive adenosine incorporation. Consistent with this regulatory mechanism, loss-of-function mutations of PAP in *Drosophila* disrupt poly(A) tail elongation and destabilize cellular mRNA populations (Juge et al., 2002). Cumulatively, PAS elements represent foundational *cis*-regulatory determinants of mRNA maturation and constitute the molecular basis of alternative polyadenylation (APA).

Most eukaryotic genes harbor multiple distinct polyadenylation cleavage sites, and APA represents a pivotal evolutionary mechanism driving transcriptome diversity. APA is defined as a context-dependent regulatory strategy, whereby individual genes deploy distinct PAS motifs at alternative cleavage positions in a tissue-specific or stimulus-dependent manner (Mitschka and Mayr, 2022). Transcriptome profiling of human and murine samples has revealed that more than 50% of mammalian mRNAs contain at least two APA-regulated PAS sites (Wang et al., 2018). The functional consequence of APA are strictly determined by the positional distribution of PASs. PASs located downstream of the stop codon generally do not alter the amino acid sequence of encoded proteins. In contrast, PASs upstream of the stop codon may lead to changes in the protein sequence or even abolish stop codon function (Hoarau et al., 2004). Despite the well-recognized role of APA as a potent layer of transcriptional gene control, the molecular mechanisms governing selective PAS site selection remains incompletely defined (Larochelle et al., 2017).

Notably, a critical unresolved gap persists regarding the mechanistic crosstalk between promoter-driven transcriptional output and downstream polyadenylation efficiency. Accumulating evidence has confirmed that transcription and 3′-end processing occur are tightly coupled co-transcriptional events, however, quantitative evidence directly linking transcriptional activity to polyadenylation output remains sparse. Nagaike et al. reported that the transcription factor GAL4-VP16 enhances polyadenylation efficiency through physical interaction with the PAF elongation complex, yet systematic quantification of how graded transcriptional activity modulates polyadenylation efficiency is still lacking (Proudfoot et al., 2002; Nagaike et al., 2011). Ji et al. previously identified a connection between transcriptional levels and APA patterns, demonstrating that highly expressed genes tend to generate transcripts with truncated 3'untranslated regions (3'UTRs) (Ji et al., 2011). Nevertheless, targeted research focusing on transcriptional regulation of individual polyadenylation sites remains insufficient.

Beyond basal regulatory mechanisms, APA undergoes dynamic remodeling throughout organismal development and cell differentiation. Conventionally, proliferative cells preferentially generate transcripts with truncated 3′UTRs (Sandberg et al., 2008), while differentiating cell lineages typically shift toward transcripts bearing longer 3′UTRs (Ji et al., 2009). Intriguingly, placental trophoblast syncytialization deviates from this canonical pattern. During gestation, mononuclear cytotrophoblasts fuse and differentiate into multinucleated syncytiotrophoblasts, a specialized secretory epithelial layer that secretes abundant hormones, growth factors, and functional proteins essential for pregnancy maintenance (Costa, 2016). This unique trophoblast differentiation process is characterized by widespread 3′UTR shortening, which is tightly coupled to the upregulation of genes associated with cellular protein secretion (Cheng et al., 2020). Despite these observed transcriptomic alterations, direct evidence linking PAS-mediated 3′-end processing decisions to trophoblast syncytialization remains scarce. Most existing studies have profiled global APA landscapes during placental development but failed to resolve the regulatory role of individual PAS elements (Tian and Manley, 2017). Furthermore, it remains uncharacterized whether sequence variations or mutations in PAS motifs alter APA isoform distribution during syncytialization or perturb the expression of core placental functional genes. This unresolved knowledge gap is particularly critical for human placentation research, as mutations in PAS hexamer motifs can redirect APA site selection, thereby profoundly altering mRNA stability and downstream protein abundance (Tian and Manley, 2017). Such PAS-dependent 3′-end processing regulation is hypothesized to be crucial for fine-tuning the expression of genes that support the robust secretory function of syncytiotrophoblasts. To address the undefined relationship between transcriptional activity and single-PAS polyadenylation efficiency, this study systematically investigated how graded transcriptional levels modulate the processing activity of individual PAS elements. Our results demonstrate that elevated transcriptional activity markedly enhances polyadenylation efficiency at single PAS sites, with canonical high-strength PASs exhibiting more pronounced responsiveness. For highly expressed genes, polyadenylation efficiency across multiple discrete PASs was found to increase synergistically. Using an *in vitro* human trophoblast syncytialization model, we further revealed that genes promoting syncytialization possess inherently robust PAS motifs, whereas genes exerting inhibitory effects on syncytialization harboring weak PASs show negligible improvements in polyadenylation efficiency under elevated transcription. Collectively, this work presents systematic, single-site-resolution evidence for the transcriptional modulation of polyadenylation at individual PAS elements. We further establish that this PAS-dependent regulatory mechanism is tightly coupled to human placental trophoblast syncytialization. These findings fill a critical gap in the mechanistic understanding of transcription-coupled mRNA processing and provide a fundamental basis for future studies exploring how PAS motifs sustain the secretory function of syncytiotrophoblasts by regulating target gene expression.

## Materials and methods

### Cell culture and transfection

HeLa cells were maintained at 37 °C in a humidified with 5% CO_2_ atmosphere using Dulbecco’s Modified Eagle’s Medium (DMEM, Hyclone) supplemented with 10% Fetal Bovine Serum (FBS; Gibco) and 1% Penicillin-Streptomycin Solution (Gibco) for routine passage and subsequent experiments.

For dual-luciferase reporter experiments, HeLa cells were seeded in 12-well plates at a density of 1 × 10^5^ cells per well and cultured for 24 h to transfection. A total of 1 μg of PAS luciferase reporter vector was transfected into each well using 2 μL of X-treme GENE HP transfection reagent according to the manufacturer’s standard protocol. Transfected cells were harvested 48 h post-transfection for subsequent quantification of luciferase activity.

Human choriocarcinoma BeWo trophoblast cells (catalog no. CCL-98) were purchased from the American Type Culture Collection (ATCC, Manassas, VA, USA). After recovery from cryopreservation, cells were cultured in a 1:1 mixture of high-glucose DMEM (Hyclone) and Ham’s F-12K medium (Kaighn’s formulation, Gibco), supplemented with 10% FBS (Hyclone), following a previously established culture protocol (Pattillo et al., 1968). During continuous passaging, *mycoplasma* contamination was routinely monitored via DAPI (Sigma) nuclear staining and phase-contrast microscopy to ensure cellular purity.

### Construction of SV40 promoter gradient deletion dual-luciferase vectors

To obtain reporter constructs with graded transcriptional activity, a series of SV40 promoter-truncated plasmids were generated based on the parental pRenilla-IRES-Firefly dual-luciferase backbone. Three constructs with sequential promoter truncations were established: a full-length SV40 promoter control (pSV40-Δ0, Control), a 100 bp truncated variant (pSV40-Δ100), and a 200 bp truncated variant (pSV40-Δ200), as illustrated in Figure 1A. The native 270 bp SV40 promoter was truncated via inverse PCR using two forward deletion primers (delta100-F and delta200-F) and a universal reverse primer (CMV-Reverse), as listed in Supplementary Table A1.

**FIGURE 1:**
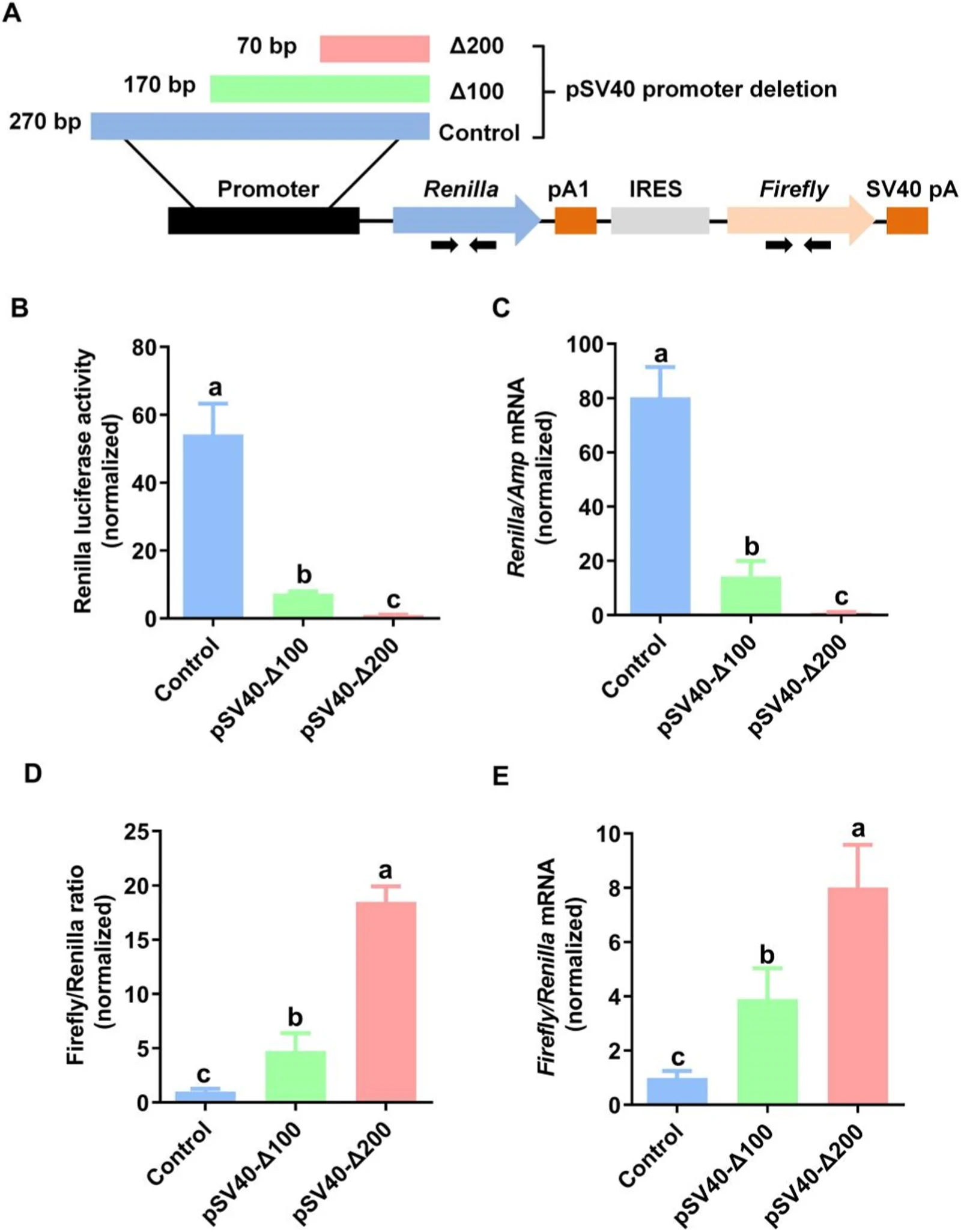
Truncation of the SV40 promoter suppresses transcriptional activity and alters relative transcript abundance from the bicistronic dual-luciferase reporter. **(A)** Schematic illustration of reporter plasmids carrying serially truncated SV40 promoter fragments. Three constructs were generated: full-length 270 bp pSV40 (Control), 170 bp (pSV40-Δ100), and 70 bp (pSV40-Δ200). Each plasmid contains a bicistronic reporter cassette composed of Renilla luciferase, an internal ribosome entry site (IRES), and Firefly luciferase. **(B)** Quantification of normalized Renilla luciferase activity shows maximal signals in the full-length control group, intermediate signals in pSV40-Δ100, and drastically diminished signals in pSV40-Δ200. **(C)** qRT-PCR quantification of relative Renilla mRNA levels recapitulates the luciferase activity profiles, confirming that promoter truncation restricts transcriptional output. **(D)** Calculated Firefly/Renilla luciferase activity ratios reach the minimum value in the control group, display moderate values in pSV40-Δ100, and peak in the pSV40-Δ200 group. **(E)** qRT-PCR-measured Firefly/Renilla mRNA ratios exhibit trends consistent with luminescence data, which reflects stable IRES-driven Firefly transcription alongside attenuated Renilla mRNA production. Raw luminescence values were first normalized to the total protein concentration of each well to eliminate deviations caused by differences in cell number and lysis efficiency **(B,D)**. All groups were then relatively quantified with the mean value of the experimental group (pSV40-Δ200) set to 1, which is the meaning of “normalized” on the Y-axis **(B)**. The ampicillin resistance gene (*Amp*) on the plasmid as the internal reference to correct for differences in plasmid copy number across samples. The relative expression of Renilla mRNA was normalized to the copy number of the *Amp* gene. All groups were then relatively quantified with the mean value of the experimental group (pSV40-Δ200) set to 1, which is the meaning of “normalized” on the Y-axis **(C,E)**. Statistically significant differences between groups (*P* < 0.05) are denoted by distinct letter labels **(a–d)**.

Inverse PCR was performed in a 50 μL reaction system containing 10 μL of 5× PrimeSTAR GXL Buffer, 1 μL of PrimeSTAR GXL DNA Polymerase (Takara), 5 μL of 2.5 mM dNTP mixture, 2 μL of forward primer, 2 μL of reverse primer, 1 μL of template plasmid (10 ng), and 39 μL of nuclease-free water. The thermal cycling program was set as follows: initial denaturation at 95 °C for 5 min; 30 cycles of 98 °C for 10 s and 60 °C for 1 min per kb; and a final extension at 68 °C for 10 min.

Purified linear PCR products were circularized by seamless homologous recombination using a Seamless Cloning Kit (Taihe Biotechnology, Beijing, China). Briefly, 50 ng of purified linear backbone was mixed with 5 μL of 2 × Seamless Master Mix and nuclease-free water to a final volume of 10 μL, followed by incubation at 50 °C for 15 min. Subsequently, the recombination products were transformed into DH5α competent *Escherichia coli* cells. Positive clones were identified by colony PCR as previously described (Deng et al., 2018), amplified in liquid culture, and validated by Sanger sequencing to obtain the final three promoter-truncated reporter constructs (pSV40-Δ0, pSV40-Δ100, pSV40-Δ200).

### Quantitative Real-Time reverse transcription PCR(qRT-PCR)

Total RNA was extracted from HeLa cells and BeWo cells using the TRIrizol reagent (Invitrogen, cat# 15596026CN) with the standard procedure of the manufacturer. First-strand cDNA was synthesized using a First Strand cDNA Synthesis Kit (Tiangen Biotech, Beijing, cat# ER103). qRT-PCR amplification was performed with the Super Real preMix Plus kit (Tiangen Biotech, Beijing, cat# FP205) on an ABI 7500 Real-Time PCR System. All primer sequences used for qRT-PCR quantification are summarized in Supplementary Table A1.

### Construction of Tet-On inducible reporter vectors and PAS mutant plasmids

For Tet-On inducible dual-luciferase vector construction, the constitutive CMV promoter of the pRenilla-IRES-Firefly backbone was replaced with a TRE-containing pTight Tet-On inducible promoter. The Tet-On promoter fragment was amplified from the pLVX-Tet-On Advanced plasmid using primer pairs TET-bglII-F/TET-nheI-R (Supplementary Table A1). The original CMV promoter was removed via reverse PCR linearization using psi-tet-CMVdel-F/psi-tet-CMVdel-R primers. The amplified promoter insert and linearized vector backbone were assembled through seamless homologous recombination under the conditions described above, followed by transformation into DH5α cells and verified by Sanger sequencing.

To generate reporter plasmids carrying distinct PAS variants, the pRenilla-IRES-Firefly vector was linearized via inverse PCR using a universal reverse primer (IRES-Reverse) and different forward primers corresponding to wild-type PAS, PAS deletion, and point-mutated PAS variants. Five distinct PAS sequences (AATAAA, ATTAAA, ACTAAA, AGTAAA, and CATAAA) were individually inserted between the Renilla luciferase coding region and the IRES element. All PCR amplification and seamless cloning procedures were performed using identical reaction systems and incubation conditions as described above.

For Tet-On induction experiments, cells transfected with Tet-On reporter constructs were treated with escalating concentrations of doxycycline (Dox; Sigma) for 24 h to induce graded transcriptional activation. Cellular luciferase activity was subsequently detected using the Dual-Luciferase Reporter Assay System (Promega).

### Dual-luciferase activity measurement

At 48 hours post-transfection, HeLa cells were washed once with pre-cooled phosphate-buffered saline (PBS) and subsequently lysed using 1 × Passive Lysis Buffer (Promega). The resulting cell lysates were collected and centrifuged at 12,000 × g for 5 min at 4 °C to eliminate insoluble cellular debris. Dual-Luciferase activity was assessed utilizing the Dual-Luciferase Reporter Assay System (Promega), following the manufacturer’s guidelines.

Briefly, a 20 µL aliquot of clarified lysate was transferred to a white opaque 96-well plate. Firefly luciferase activity was measured immediately after the addition of 100 µL Luciferase Assay Reagent II, and Renilla luciferase activity was quantified following the addition of 100 μL Stop & Glo® Reagent. Luminescence signals were captured using a GloMax® Navigator Microplate Luminometer.

All dual-luciferase assays were performed with equal plasmid transfection dosage across experimental groups. The bicistronic vector design enables simultaneous expression of Firefly and Renilla luciferases from a single transcript. Renilla luciferase activity was used as an internal control to normalize variations in transfection efficiency and plasmid dosage.

### Immunofluorescence and confocal microscopy

BeWo cells were fixed in pre-chilled methanol for 10 min at room temperature. Following thorough rinsing with PBS, cells were incubated overnight at 4 °C with primary antibody against human E-cadherin (Cell Signaling Technology, #3195). After extensive PBS washing, cells were exposed to TRITC 594-conjugated anti-rabbit IgG secondary antibodies (ZhongShan Golden Bridge) for 45 min at room temperature. Negative control samples were processed in parallel by replacing the primary antibody with equal concentrations of non-specific rabbit IgG (Abcam) to validate staining specificity. Cellular nuclei were subsequently counterstained with DAPI. Fluorescence images were finally acquired using a ZEISS LSM 780 laser scanning confocal microscope.

### Western blotting

Total cellular protein was extracted from BeWo cells using freshly prepared RIPA lysis buffer containing 25 mM Tris-HCl (pH 7.4), 150 mM NaCl, 0.1% SDS, 1% NP-40, 1 mM EDTA and 1 mM sodium orthovanadate, supplemented with a protease inhibitor cocktail (Sigma-Aldrich) immediately before use. Cell lysates were centrifuged at 10,000 × g for 10 min at 4 °C, and supernatants free of insoluble debris were retained for downstream quantification. Total protein concentration was quantified using a BCA Protein Assay Kit (Beyotime Biotechnology) strictly as outlined in the supplier’s specifications.

For electrophoretic separation, a total of 30 μg protein per sample was loaded onto 12% SDS-containing polyacrylamide gels, and electrotransferred onto nitrocellulose membranes (Millipore). Membranes were blocked with 5% BSA (Sigma) diluted blocking solution to eliminate non-specific antibody binding, followed by overnight incubation with primary antibodies at 4 °C: anti-hCGβ (Abcam, Ab9582) and anti-HERV-FRD (Abcam, Ab171382) and HRP-tagged secondary antibodies (Abcom). Chemiluminescent bands were developed using ECL substrates (Amersham Life Science) and captured on the GeneGnome XRQ imaging platform (Syngene). Band intensity was quantified using Quantity One® v4.4 software (Bio-Rad), and β-actin was used to normalise the relative abundance of target proteins.

### Quantification of polyadenylation efficiency

BeWo cell syncytialization was induced by forskolin (FSK) treatment to activate the cAMP signaling pathway. Total RNA was extracted from FSK-stimulated and DMSO control BeWo cells using TRIzol reagent according to the manufacturer’s instructions, and cDNA was synthesized following previously established protocols. qRT-PCR primers were designed to target upstream and downstream regions flanking the mRNA 3′-end cleavage and polyadenylation site (Supplementary Figure A1). All qRT-PCR experiments were performed in three independent biological replicates.

Polyadenylation efficiency was quantified using a modified 2^−ΔΔCT^ method. The ΔCt value was defined as the Ct difference between target amplicons and reference genes, and the relative expression (RE) of each amplicon was calculated as 2^−ΔΔCT^. The regional expression level (REL) was determined as the ratio of downstream RE to upstream RE. Normalized polyadenylation efficiency was finally calculated as the ratio of FSK-treated REL to DMSO control REL. All results are presented as mean ± standard deviation (SD) from no fewer than three independent biological replicates.

### Statistical analysis

All experimental data were presented as the mean ± SD from a minimum of three independent experiments. Statistical analyses were conducted using GraphPad Prism 9.0 software. To check the significance of mean value differences, one-way ANOVA followed by Tukey’s honest significance test were performed for multiple comparisons in Figures 1–3, and statistically significant differences (*P* < 0.05) were marked with different letters (a–d). For two-group comparisons in Figure 4, unpaired Student’s t-test was used, with statistical significance defined as *p < 0.05.

**FIGURE 2:**
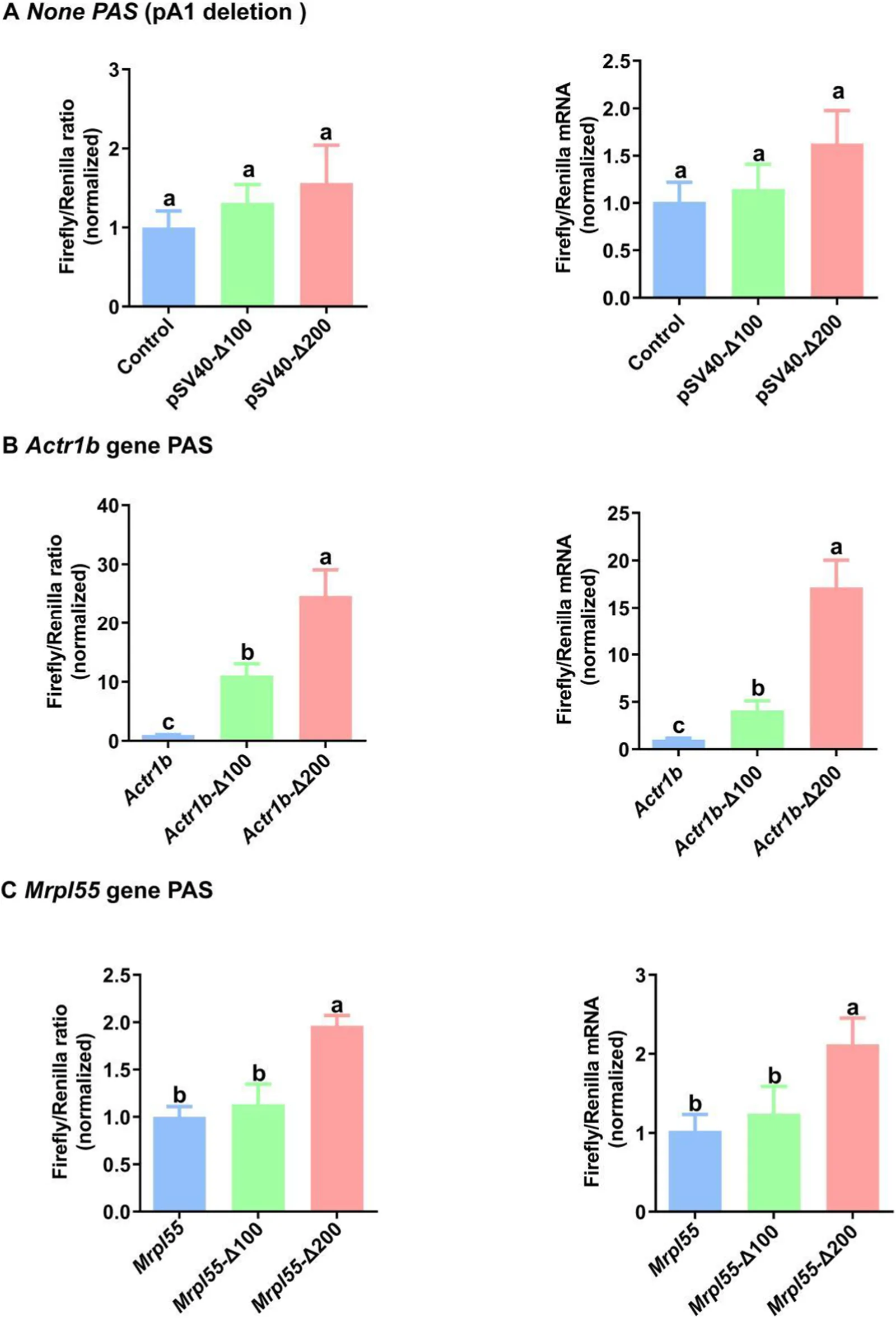
Distinct endogenous polyadenylation signal sequences determine how SV40 promoter truncation modulates the expression patterns of bicistronic reporter transcripts. **(A)** Reporter construct lacking the upstream pA1 polyadenylation signal. Firefly/Renilla ratios determined by luciferase activity (left panels) and mRNA quantification (right panels) exhibited no statistically significant differences across control, pSV40-Δ100, and pSV40-Δ200 groups, which were uniformly marked with the identical letter label “a”. **(B)** Reporter construct carrying the endogenous PAS derived from the *Actr1b* gene. Measured Firefly/Renilla ratios (luciferase activity, left; mRNA levels, right) reached the minimum value in the full-length *Actr1b* group (labeled “c”), rose moderately in *Actr1b*-Δ100 (labeled “b”), and peaked within the *Actr1b*-Δ200 group (labeled “a”). **(C)** Reporter construct containing the endogenous PAS from the *Mrpl55* gene. Firefly/Renilla ratios (luciferase activity, left; mRNA levels, right) were comparable between the full-length *Mrpl55* and *Mrpl55*-Δ100 groups (both labeled “b”), whereas a prominent elevation was detected in the *Mrpl55*-Δ200 group (labeled “a”). Statistically significant differences between groups (*P* < 0.05) are denoted by distinct letter labels **(a–d)**.

**FIGURE 3:**
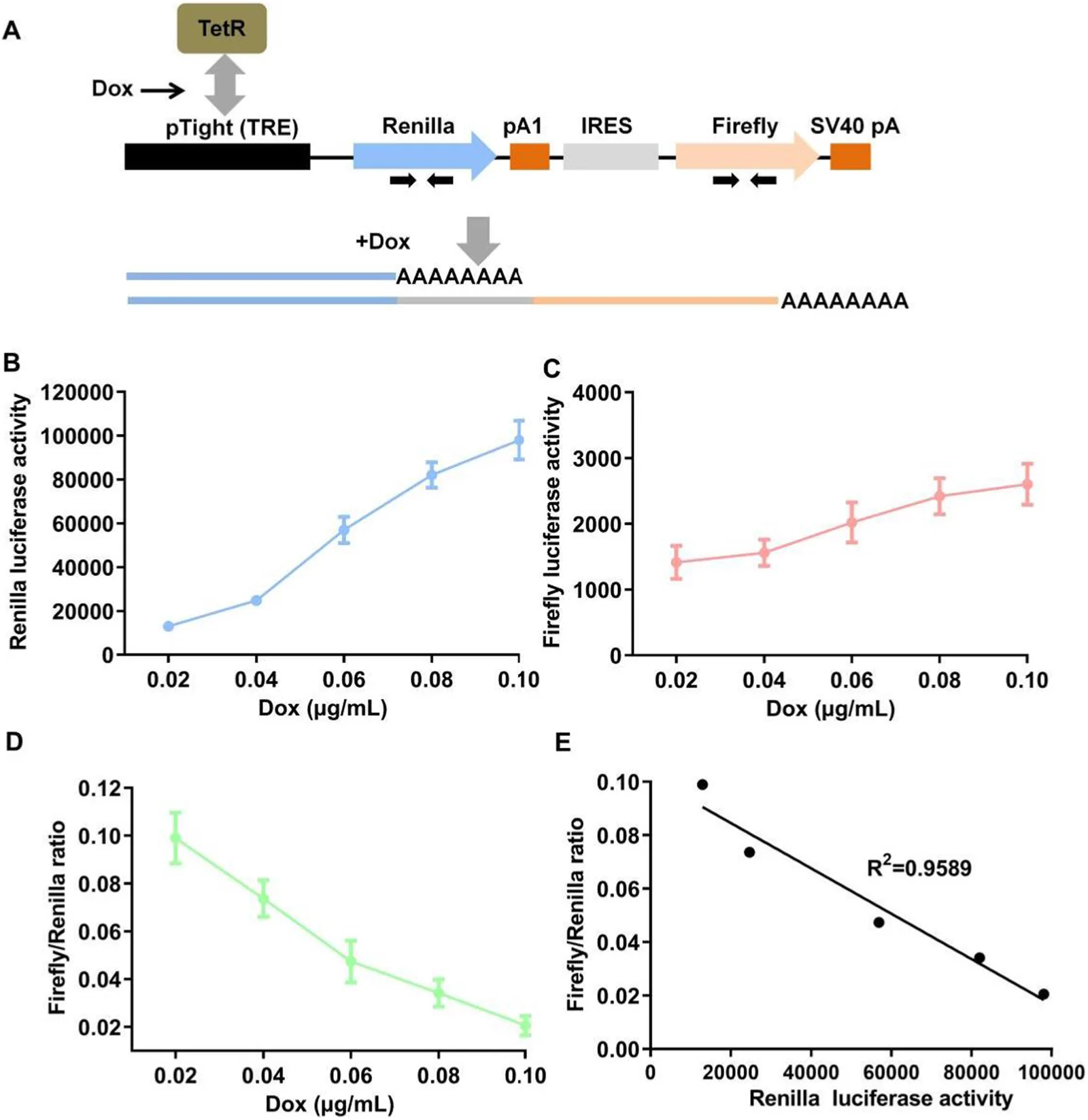
Dox-modulated transcriptional activity reshapes Firefly/Renilla expression ratio of bicistronic dual-luciferase reporters. **(A)** Schematic diagram of the Tet-On dual-reporter system. The pTight TRE promoter is constitutively inhibited by TetR, while Dox treatment relieves this repression to initiate Renilla luciferase transcription. Firefly luciferase is transcribed from the same bicistronic transcript via IRES. Enhanced transcription triggered by Dox is visualized as elongated poly **(A)** tails. **(B)** Quantification of Renilla luciferase activity reveals a dose-dependent elevation with increasing Dox concentrations. **(C)** Measurement of Firefly luciferase activity exhibits mild, gradual upregulation following Dox stimulation. **(D)** Calculated Firefly/Renilla ratios decline progressively upon elevated Dox doses, a phenomenon driven by robust transcriptional induction of Renilla luciferase. **(E)** Linear regression analysis confirms a prominent negative linear correlation between Renilla luciferase activity and Firefly/Renilla ratios (R^2^ = 0.9589).

**FIGURE 4:**
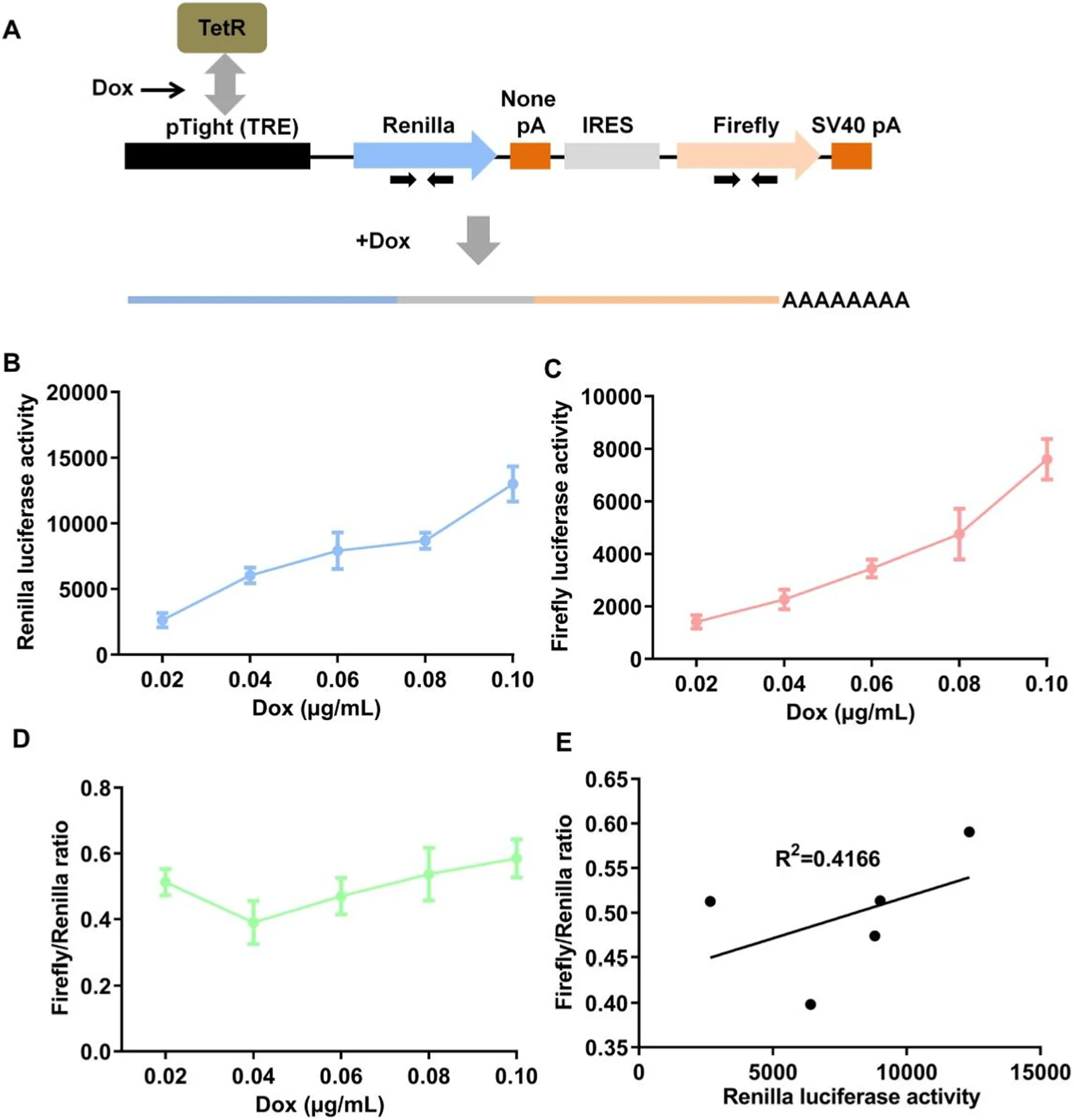
Dox-induced transcriptional responses are modified under exclusive functionality of the SV40 PAS in bicistronic reporter constructs. **(A)** Schematic diagram of the Tet-inducible dual-luciferase reporter system. The pTight (TRE) promoter is suppressed by TetR in the absence of Dox and activated upon Dox treatment to drive Renilla luciferase expression. Firefly luciferase is co-expressed from the same transcript via IRES. Elevated transcription triggered by Dox is illustrated by elongated poly **(A)** tails. **(B)** Quantification of Renilla luciferase activity displays a dose-dependent elevation alongside increasing Dox concentrations. **(C)** Measurement of Firefly luciferase activity reveals mild, gradual upregulation following Dox stimulation. **(D)** Calculated Firefly/Renilla ratios decline with rising Dox doses, which is attributed to the robust induction of Renilla luciferase transcription. **(E)** Linear regression analysis identifies a modest correlation between Renilla luciferase activity and Firefly/Renilla ratios (R^2^ = 0.4166).

## Results

### Stepwise SV40 promoter deletion attenuates transcription and modulates PAS processing efficiency in the dual-luciferase reporter system

We aimed to clarify the functional correlation between promoter-driven transcriptional activity and the relative expression of tandemly arranged genes using a dual-luciferase reporter system. To achieve this, we constructed a series of SV40 promoter variants with gradually weakened transcriptional activity by introducing sequential truncations in the full-length 270 bp SV40 promoter, including a 100 bp deletion (pSV40-Δ100) and a 200 bp deletion (pSV40-Δ200), with the intact full-length promoter serving as the control (control; Figure 1A). Subsequently, we employed dual-luciferase activity detection and qRT-PCR assays to systematically evaluate how graded promoter activity influences the expression of upstream Renilla luciferase and downstream IRES-driven Firefly luciferase.

Our results revealed that pSV40 transcriptional activity decreased sharply with increasing deletion length, the full-length promoter control group exhibited maximal Renilla luciferase expression, whereas pSV40-Δ100 showed drastically reduced signals, and pSV40-Δ200 showed near-complete ablation of Renilla expression at both the protein and mRNA levels (Figures 1B,C). Notably, the Firefly/Renilla luciferase ratio, which reflects the relative abundance of the downstream reporter transcripts, exhibited a negative correlation with promoter activity. The ratio was lowest in the full-length promoter group, moderately elevated in the pSV40-Δ100 group, and further increased in the pSV40-Δ200 group (Figures 1D,E). This consistent trend indicated that IRES-mediated Firefly expression remained relatively stable, whereas transcriptional output of the upstream Renilla gene was gradually repressed alongside promoter truncation.

Quantitative analysis further verified this regulatory pattern. Compared with the full-length control, the Firefly/Renilla ratio increased approximately four-fold in the pSV40-Δ100 group and eight-fold in the pSV40-Δ200 group (Figure 1E). Collectively, these findings demonstrate that truncation of the pSV40 promoter directly modulates the transcription of the upstream gene, thereby altering the relative abundance of downstream transcripts within this bicistronic reporter construct.

### Functional PAS is indispensable for promoter-dependent remodeling of reporter expression balance

To determine whether PAS integrity is required for promoter-mediated modulation of gene expression ratios, we quantified Firefly/Renilla ratios at both the protein and transcript levels under three distinct PAS configurations. In PAS-deficient reporter constructs with deleted pA1 elements (Figure 2A), stepwise promoter truncation failed to significantly alter the Firefly/Renilla ratios at either the protein or mRNA level, with all groups showing statistically indistinguishable values (group ‘a’). This observation indicates that changes in promoter activity alone cannot reshape the relative expression of tandem reporter genes in the absence of a functional upstream PAS.

We next evaluated two endogenously encoded human PAS elements with well-defined activities and sequence characteristics to further dissect the crosstalk between transcription strength and polyadenylation efficiency. The 3'UTR of *Actr1b* harbors the canonical AAUAAA hexamer, a highly conserved that drives robust and efficient polyadenylation. In contrast, the 3'UTR of *Mrpl55* contains a single-nucleotide variant AUUAAA, a common non-canonical PAS with significantly reduced activity relative to AAUAAA. By comparing these strong and weak endogenous PAS elements, we aimed to define the functional coupling between transcription and mRNA 3'-end polyadenylation.

In constructs carrying the strong canonical *Actr1b* PAS, the Firefly/Renilla ratio was minimal under the full-length promoter (group ‘c’), moderately elevated following 100 bp deletion (group ‘b’), and sharply increased upon 200 bp truncation (group ‘a’) (Figure 2B). A similar but attenuated trend was observed for the weaker non-canonical *Mrpl55* PAS: the Firefly/Renilla ratios remained comparable between the full-length and Δ100 promoter groups (both group ‘b’), but were significantly enhanced in the Δ200 truncation mutant (group ‘a’) (Figure 2C). Taken together, these data demonstrate that promoter truncation preferentially boosts relative downstream reporter expression in the presence of a functional PAS, whereas this regulatory effect is fully abolished in PAS-deficient contexts.

### Establishment of a Tet-On inducible reporter system for dynamic Single-PAS function analysis

To validate the above static truncation-based findings under dynamically tunable transcriptional conditions, we engineered a Dox-inducible dual-luciferase reporter system by replacing the constitutive CMV promoter with a Tet-On-responsive pTight promoter. We then generated a modified bicistronic construct contains a tetracycline response element (TRE) and orthogonal reporter architecture, dose-dependent transcriptional activation in response to Dox treatment. In this system, the pTight promoter drives the transcription of a single bicistronic mRNA, in which Renilla luciferase is expressed as the upstream gene, and Firefly luciferase is translated via an IRES element, with the SV40 late polyadenylation signal positioned at the 3′terminus (Figure 3A). In the absence of Dox, the tetracycline repressor (TetR) occupies the pTight promoter and effectively suppresses basal transcription. Dox administration triggers TetR dissociation from the promoter, thereby initiating robust transcription of bicistronic transcripts that undergo canonical PAS-mediated 3′-end processing.

We measured dual-luciferase activities across a gradient of Dox concentrations (0.02–0.10 μg/mL) in the above system. Renilla luciferase activity increased sharply in response to Dox, reaching approximately 100,000 relative light units at the highest Dox concentration (0.10 μg/mL; Figure 3B). By comparison, Firefly luciferase activity increased gradually with Dox induction but displayed much slower response kinetics (Figure 3C). Consistent with this differential activation pattern, the Firefly/Renilla ratio decreased progressively with increasing Dox dosage (Figure 3D). Linear correlation analysis confirmed a strong negative association between total transcriptional output (reflected by Renilla activity) and the Firefly/Renilla ratio (R^2^ = 0.9589; Figure 3E), supporting a direct positive coupling between transcriptional activity and PAS-dependent 3'-end processing efficiency.

### Single functional SV40 PAS reshapes Dox-dependent expression dynamics in the Tet-On reporter system

To further isolate the regulatory function of a single defined PAS in transcription-coupled processing, we optimized the Tet-On reporter framework to retain only one functional SV40 PAS (Figure 4A). In this streamlined system, the pTight promoter controls bicistronic transcription, with Renilla expressed upstream and IRES-driven Firefly expressed downstream from the same transcript. Basal transcription is tightly repressed by TetR, while Dox induction efficiently activates transcriptional initiation and full-length mRNA synthesis.

Under Dox induction, Renilla luciferase activity increased in a clear dose-dependent manner (Figure 4B), and Firefly luciferase activity rose in parallel, with a more pronounced induction at higher Dox concentrations (Figure 4C). In contrast to the previous dual-PAS reporter system, the Firefly/Renilla ratio exhibited only a modest overall increase with rising Dox levels (Figure 4D), yielding a moderate positive correlation with Renilla transcriptional activity (R^2^ = 0.4166; Figure 4E). These results indicate that the strong inverse relationship observed in conventional reporter systems is strongly modulated by PAS architecture and becomes substantially blunted when only a single downstream SV40 PAS is operational.

### Distinct PAS variants generate divergent reporter outputs under uniform transcriptional induction

To eliminate transcriptional variability and directly compare the intrinsic processing activity of different PAS sequences, we performed reporter assays under unified transcriptional input. HeLa Tet-On cells were transfected with reporters carrying five prevalent PAS variants (AATAAA, ATTAAA, ACTAAA, AGTAAA, or CATAAA), which were individually inserted between the Renilla coding sequence and the downstream IRES-Firefly cassette, followed by treatment with a fixed Dox concentration of 0.05 μg/mL to achieve consistent transcriptional activation.

At standardized induction conditions, PAS identity strongly influenced the Firefly/Renilla reporter ratios (Figures 5B,C). The canonical AATAAA motif yielded the lowest Firefly/Renilla ratio, reflecting highly efficient recognition and processing of this strong PAS. In contrast, ATTAAA and ACTAAA variants displayed intermediate ratios, whereas AGTAAA and CATAAA produced the highest ratios, indicative of impaired PAS function. These results demonstrate that, even under equivalent transcriptional conditions, inherent PAS sequence strength is a dominant determinant of 3′-end processing efficiency and bicistronic reporter partitioning.

**FIGURE 5:**
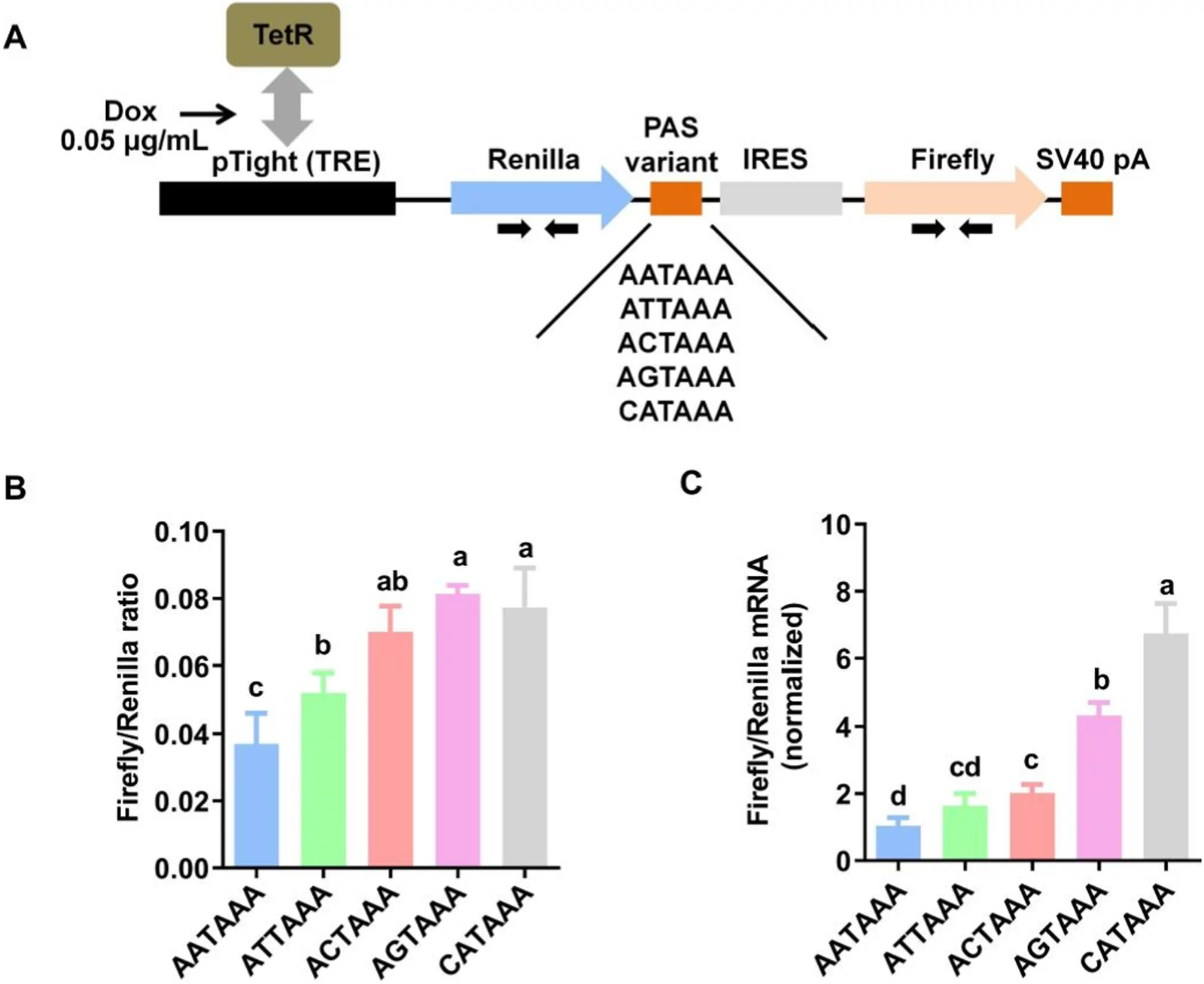
Dox-triggered transcription generates divergent Firefly/Renilla expression ratios determined by distinct PAS hexamer variants in HeLa reporter cells. **(A)** Schematic diagram of the Dox-inducible bicistronic reporter construct. The Renilla luciferase cistron is under the control of the pTight/TRE promoter, followed by individual PAS hexamer variants, an IRES element, the Firefly luciferase cistron, and a terminal SV40 PAS. Transcriptional induction was induced with 0.05 μg/mL Dox. The tested PAS variants include the canonical AATAAA and four non-canonical variants: ATTAAA, ACTAAA, AGTAAA, and CATAAA. **(B)** Quantification of normalized Firefly/Renilla luciferase activity ratios demonstrates expression patterns shaped by PAS identity. **(C)** qRT-PCR measurement of Firefly/Renilla mRNA ratios recapitulates the regulatory trends observed in luciferase activity assays. Statistically significant differences between groups (*P* < 0.05) are denoted by distinct letter labels (a–d).

### Elevated ttranscription preferentially potentiates processing of high-strength PAS variants

We next examined how graded transcriptional activation differentially modulates the processing efficiency of distinct PAS variants across Dox dose-gradient induction. All five PAS-containing reporter constructs displayed increasing Renilla expression in response to rising Dox concentrations, confirming consistent and uniform activation of the Tet-On promoter across all experimental groups (Figure 6, left panels). However, Firefly/Renilla ratio responded differentially in a PAS-dependent manner (Figure 6, right panels).

**FIGURE 6:**
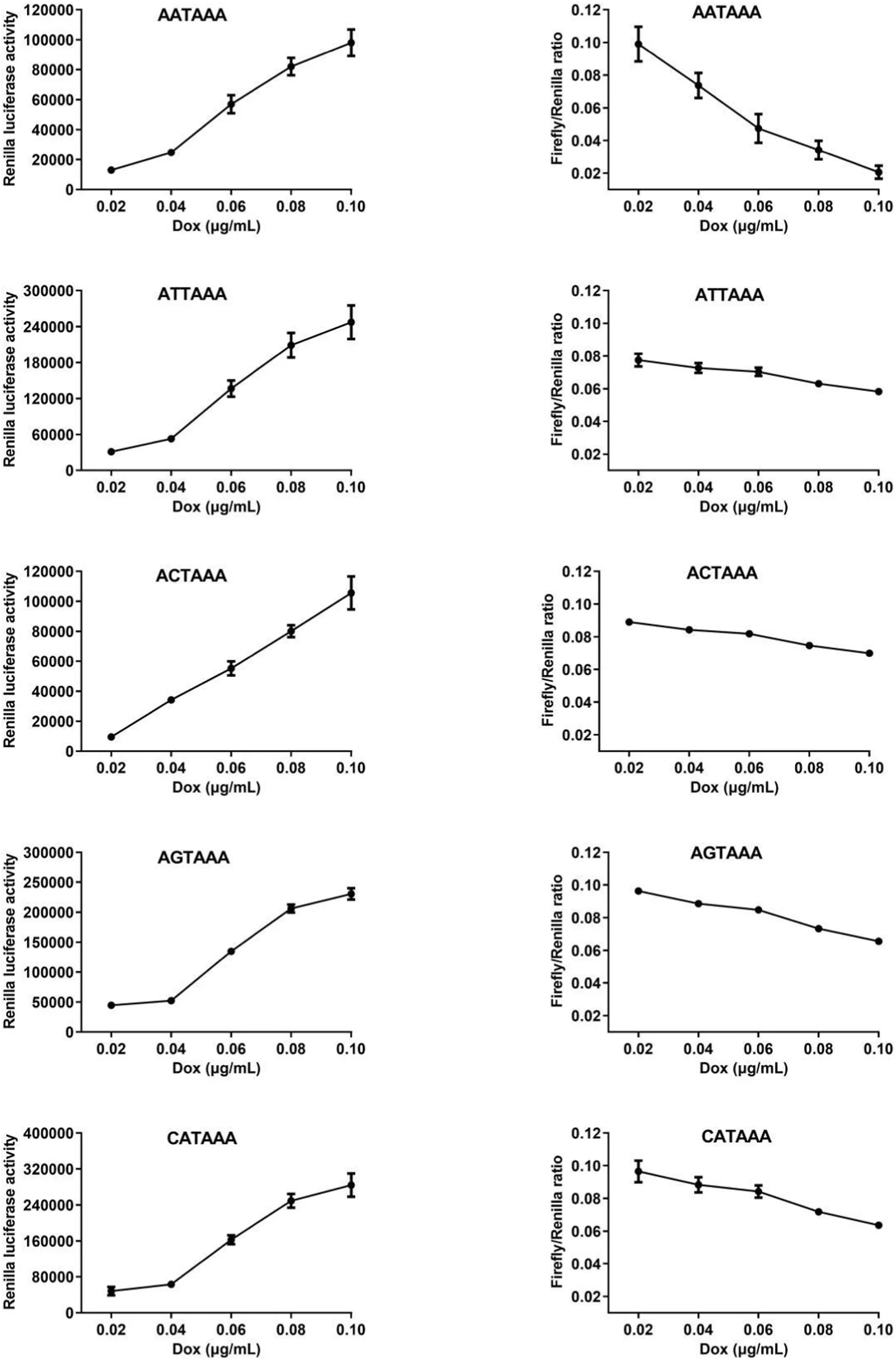
Different PAS variants exhibit divergent dose-dependent responses to graded transcriptional activation. Left panels: Renilla luciferase activity (y-axis) displays a gradual upward trend across escalating Dox concentrations (0.02–0.10 μg/mL, x-axis) for each tested PAS variant (AATAAA, ATTAAA, ACTAAA, AGTAAA, CATAAA). Right panels: Normalized Firefly/Renilla ratio (normalized to the value measured at 0.10 μg/mL Dox) reveal a pronounced reduction for the AATAAA motif upon increasing Dox doses. All remaining PAS variants only underwent mild fluctuations in their ratio values. All data are presented as mean ± SD from no fewer than three independent experiments.

The canonical AATAAA motif exhibited the most dramatic reduction in Firefly/Renilla ratio with increasing Dox dosage, implying that its polyadenylation efficiency is most strongly enhanced under conditions of maximal transcriptional output. The ATTAAA variant exhibited an intermediate response to elevated transcription, whereas ACTAAA, AGTAAA, and CATAAA variant generally maintained relatively stable ratios across the entire Dox concentration range. These results reveal a preferential regulatory pattern: enhanced transcriptional activity selectively boosts the processing efficiency of potent canonical PAS elements, while weak non-canonical PASs are largely insensitive to transcriptional elevation. The magnitude of this transcription-coupled regulatory effect is directly determined by the intrinsic biochemical activity of individual PAS sequences.

### Canonical PASs of trophoblast fusion genes are selectively mobilized during FSK-Induced BeWo cell syncytialization

To validate our cellular and molecular findings in a physiological context, we adopted an established *in vitro* model of human trophoblast syncytialization via FSK stimulation of BeWo cells. FSK stimulation successfully triggered obvious cell fusion and multinucleated syncytiotrophoblast formation (Figures 7A,B). Consistent with these morphological changes, the protein abundance of core syncytial markers β-hCG and syncytin2 were significantly upregulated at 24 h and 48 h post-treatment relative to untreated controls, verifying the successful establishment of the BeWo syncytialization model (Figures 7C–E).

**FIGURE 7:**
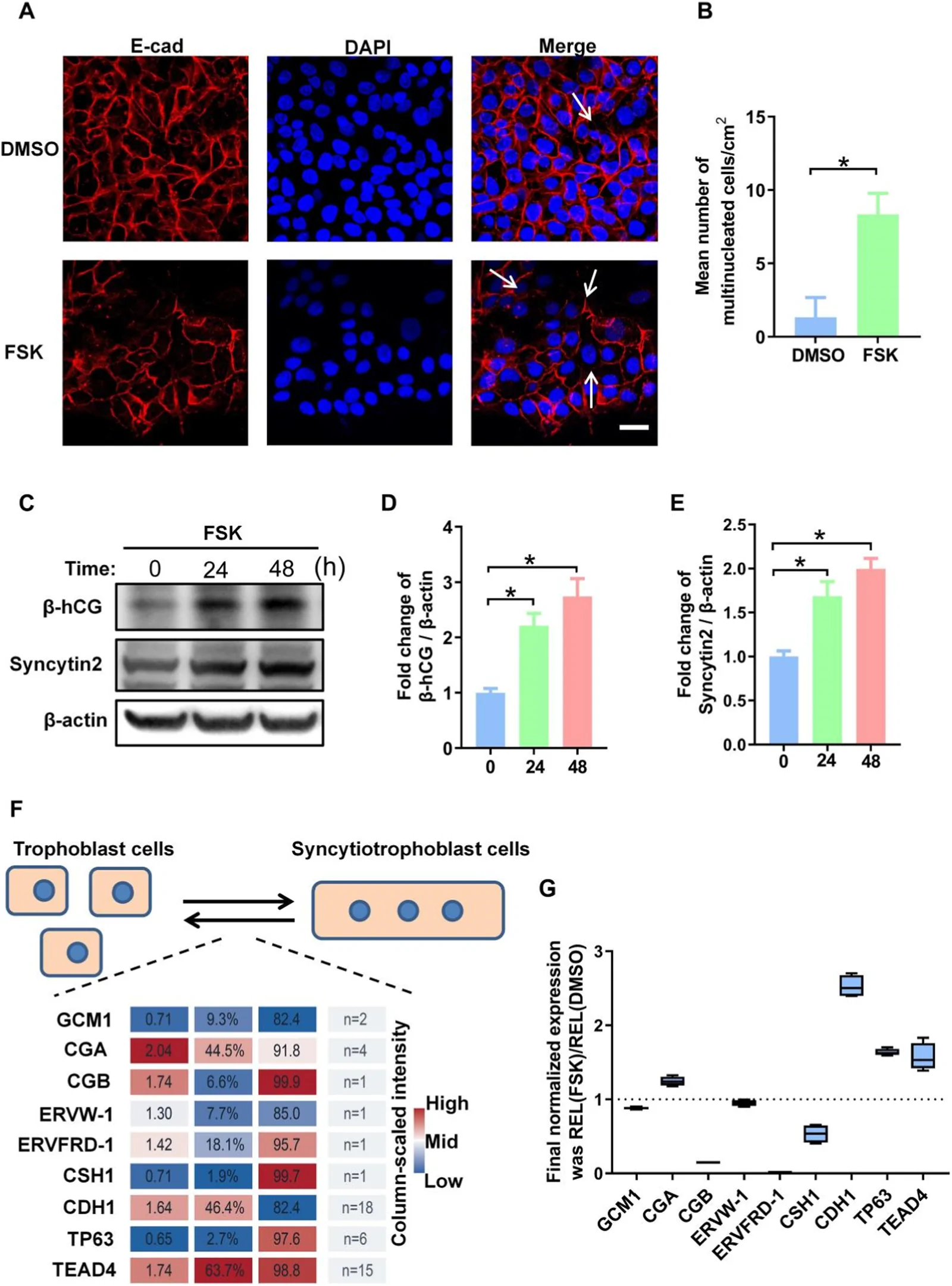
FSK-mediated trophoblast syncytialization promotes the utilization of high-activity PAS isoforms through transcriptional upregulation in BeWo cells. **(A)** Immunostaining of E-cadherin (red) and DAPI (blue) in FSK-treated BeWo cells. White arrows indicate multinucleated cells with two or more nuclei. Scale bars, 50 μm. **(B)** The numbers of multinucleated cells in each group are quantified in five random fields and recorded as cell number/cm^2^. **(C–E)** Typical results **(C)** and statistical analysis **(D,E)** of western blotting for β-hCG and Syncytin2 in BeWo cells with 20 μM FSK for 24 h or 48h. **(F)** PAS annotations were retrieved from the human hg38 Main collection of PolyA_DB v4.1. PAS activity was calculated as log10(AvgRPM.3′READS +1) from the highest AvgRPM.3′READS PAS per gene (Numerical values listed in the first column). Sample support (Numerical values listed in the second column) and PAS sequence strength (Numerical values listed in the third column) are shown as PSE.3′READS and PolyaStrength_percentile, respectively. Colours were scaled within each metric column, blue, white and red indicate low, intermediate and high values. Cell labels indicate original unscaled values, and the grey column shows the number of main collection PASs per gene. **(G)** qRT-PCR was performed to detect the relative expression of upstream and downstream fragments of the target gene from FSK-induced BeWo cells, using an internal reference gene for normalization. Expression levels were calculated using a modified 2^−ΔΔCT^ method. Regional expression level (REL) was defined as the ratio of downstream to upstream expression, and final normalized values were expressed as REL_FSK_/REL_DMSO_. Data are presented as means ± SEM based on three independently repeated experiments. The significance of differences between two treatments was analyzed by *t*-tests, *, *p* < 0.05.

We next selected nine core regulatory genes governing trophoblast syncytialization, including six pro-syncytial factors (G*CM1, CGA, CGB, ERVW-1, ERVFRD-1, CSH1*) and three anti-syncytial regulators (*CDH1, TP63*, *TEAD4*). We retrieved the PAS annotations covering sequence features, functional activity and sample support of these genes from the PolyA_DB v4 database (https://tools.biociphers.org/apa-db/index). Database analysis confirmed that most pro-syncytial trophoblast genes harbor the canonical AATAAA PAS, while ERVW-1 uniquely carries the moderate-activity ATTAAA variant (Figure 7F).

We subsequently quantified the endogenous polyadenylation efficiency of these genes via qRT-PCR using primer pairs flanking their 3′-end cleavage sites. Polyadenylation efficiency was calculated as the expression ratio of downstream amplicons to upstream amplicons of each target transcript. Pro-syncytial genes exhibited downstream/upstream expression ratios close to or marginally higher than 1, indicating efficient 3′-end polyadenylation. In contrast, anti-syncytial genes displayed significantly higher expression ratios, reflecting impaired PAS processing (Figure 7G). These endogenous validation results support a model in which highly expressed pro-syncytial genes are equipped with intrinsically strong PAS elements, whereas anti-syncytial genes rely on weaker PAS signals that are less responsive to elevated transcriptional output.

## Discussion

Transcriptional initiation and 3′-end mRNA polyadenylation are two tightly interconnected processes that dictate gene expression outcomes in eukaryote cells. Current studies has predominantly focused on promoter-terminator looping and chromatin spatial crosstalk to elucidate how genomic architecture modulates the entire transcriptional cycle (Ansari and Hampsey, 2005; Lainé et al., 2009; Lamas-Maceiras et al., 2016; O'Sullivan et al., 2004; Tan-Wong et al., 2012). However, these genome-wide investigations predominantly characterize the global coordinated regulatory patterns of endogenous full-length genes, while largely neglecting how graded transcriptional activity quantitatively fine-tunes polyadenylation efficiency at individual PAS. Furthermore, it remains poorly understood whether PAS variants with distinct intrinsic activities exhibit differential sensitivities to dynamic transcriptional fluctuations. In this study, we addressed these critical research gaps by systematically dissecting the cooperative effects of transcriptional magnitude and PAS sequence identity on 3′mRNA processing efficiency. We further identify a functional association between PAS-dependent endogenous gene regulation and FSK-induced syncytialization in BeWo trophoblast cell.

We established a definitive quantitative correlation between transcriptional activity and PAS-dependent transcriptional regulation using static promoter-truncated and dynamic Tet-On inducible bicistronic reporter systems. Serial truncation of the SV40 promoter reduced the transcription of upstream Renilla luciferase in a length-dependent manner, while the expression of downstream IRES-driven Firefly luciferase remained relatively constant. This discrepancy generated a negative correlation between promoter strength and the Firefly/Renilla expression ratio. Notably, deletion of the core PAS element completely abolished this transcription-dependent cistron imbalance. This result indicates that functional PAS sequences serve as essential molecular mediators that convert promoter-originated regulatory signals to modulate the stoichiometric balance of bicistronic transcripts.

Dox dose-gradient induction further uncovered divergent responses of distinct PAS hexamer motifs to incremental transcriptional elevation. Canonical and suboptimal strong PAS motifs (AATAAA, ATTAAA) sustained stable polyadenylation efficiency and showed low sensitivity to transcriptional variations. In contrast, weak non-canonical PAS variants (ACTAAA, AGTAAA, CATAAA) displayed markedly reduced 3′processing efficiency under enhanced transcriptional activity. These results uncover a previously unrecognized hierarchical regulatory paradigm, in which the intrinsic strength of PAS sequences dictates the adaptive amplitude of 3′mRNA processing in response to transcriptional fluctuations.

Most importantly, we determined the existence of an association between PAS-dependent endogenous gene regulation and FSK-induced BeWo cell syncytialization. Successful trophoblast syncytial differentiation was verified by robust cell fusion events and significant upregulation of the key differentiation markers β-hCG and Syncytin2. We further quantified endogenous polyadenylation efficiency of nine core trophoblast regulatory genes based on PolyA_DB v4.1 annotation and qRT-PCR. Consistent physiological regulatory patterns were observed across all tested genes. Specifically, pro-syncytialization genes predominantly contain high-activity canonical PAS motifs, which support stable and efficient 3′processing during differentiation-triggered transcriptional activation. Conversely, genes that inhibit syncytialization exhibit negligible alterations in response to transcriptional upregulation. Our findings demonstrate that high-activity PAS motifs may be involved in the transcriptional activation during the FSK-induced BeWo cell syncytialization.

Notably, our current data do not confirm a direct causal relationship between PAS-dependent regulation and trophoblast syncytialization. Synthetic reporter experiments demonstrate that PAS sequences can modulate 3′-end mRNA processing at the molecular level, whereas the BeWo cell differentiation analyses reveal a physiological association between PAS-dependent endogenous gene regulation and the syncytial state. These two lines of evidence operate at different conceptual levels: reporter experiments validate the biochemical feasibility of the proposed mechanism, whereas cell-based assays confirm its functional involvement in trophoblast differentiation. Although our cellular data cannot prove that the transcription–polyadenylation axis directly drives cell fusion, they support the activation of this regulatory axis during syncytial maturation. In line with our findings, Cheng et al. (2020) reported that syncytiotrophoblast differentiation in both BeWo cells and primary human placental cytotrophoblasts is accompanied by widespread 3′UTR shortening and intronic polyadenylation. These APA alterations are tightly coupled with the upregulation of protein secretion-related genes, a phenomenon termed secretion-coupled APA (SCAP). Collectively, these observations support a model wherein elevated transcriptional activity during syncytiotrophoblast maturation enhances 3′-end processing at proximal or high-strength poly(A) sites, thereby stabilizing and amplifying transcripts that sustain the secretory and endocrine functions of syncytiotrophoblasts. Although the transcription–polyadenylation regulatory axis in syncytiotrophoblasts remains understudied, existing evidence corroborates its activation during trophoblast differentiation, particularly in modulating cell secretory phenotypes. Nevertheless, whether this axis directly mediates cell–cell fusion remains unresolved. Future studies incorporating functional perturbation of polyadenylation factors or endogenous poly(A) signals in primary trophoblasts, combined with quantitative evaluation of cell fusion efficiency and transcriptome-wide PAS usage profiling, are required to establish definitive causal relationships.

Promoter truncation can only alter bicistronic expression ratios exclusively in the presence of intact functional PAS. Deletion of the upstream pA1 polyadenylation motif eliminated significant differences in Firefly/Renilla ratios among full-length, Δ100, and Δ200 promoter-truncated groups. This indicates that PAS acts as an essential intermediates that transduce promoter regulatory signals to balance tandem cistron expression, consistent with their canonical functions in facilitating transcription termination and mature mRNA stabilization. Functional PAS mediates efficient 3′cleavage and polyadenylation of bicistronic transcripts to tune the stoichiometry of upstream and downstream protein products (Greger and Proudfoot, 1998). Disrupted PAS activity impairs mRNA maturation and causes indiscriminate degradation of transcripts from both cistrons, eliminating all transcription-dependent expression differences (Kazerouninia et al., 2010). Our findings are consistent with previous studies demonstrating that defective PAS function uncouples transcriptional initiation from downstream RNA processing, leading to dysregulated gene expression (Dichtl et al., 2002).

We further validated this transcription-bicistronic expression relationship across distinct promoter systems using the Tet-On inducible platform. Gradual increases in Dox concentration proportionally elevated pTight/TRE-driven Renilla luciferase transcription, while Firefly luciferase expression increased modestly, thereby dynamically altering Firefly/Renilla expression ratios. Vectors with intact SV40 PAS displayed a strong negative linear correlation between Renilla activity and Firefly/Renilla ratios (R^2^ = 0.9589), supporting the model that enhanced upstream transcription preferentially shifts transcript abundance toward the upstream cistron. In contrast, constructs carrying an isolated SV40 PAS only yielded a moderate correlation (R^2^ = 0.4166), demonstrating that the genomic context of PAS remodels inter-cistron regulatory coordination and highlights layered transcriptional control of bicistronic RNAs (Jean et al., 2008). The Tet-On system enables precise and tunable transcriptional induction and has been widely utilized to dissect dynamic crosstalk between transcription and RNA maturation (Huang et al., 2010).

A critical observation is the divergent polyadenylation responsiveness to elevated transcription among distinct PAS hexamers. Canonical AATAAA and suboptimal ATTAAA motifs exhibited limited efficiency suppression within a narrow window as Dox doses (and transcription levels) rose. Conversely, non-canonical motifs (ACTAAA, AGTAAA, CATAAA) exhibited steady linear losses in 3′processing efficiency alongside transcriptional upregulation, while the inactive PAS control group produced nearly unchanged processing activity. Overall, transcriptional magnitude and PAS primary sequence jointly shape 3′processing robustness, with canonical PAS hexamers withstand transcriptional shifts far better than sensitive non-canonical counterparts (Beaudoing et al., 2000). Consistent with existing models, elevated transcription remodels the recruitment kinetics of core polyadenylation complexes including CPSF and CstF at PAS sites (Nagaike et al., 2011). The variable intrinsic affinity of distinct PAS variants for polyadenylation machinery accounts for their distinct transcriptional sensitivity, which serves as a primary driver of global 3′end processing efficiency (Yao et al., 2012). IRES-mediated downstream translation is known to operate independently of upstream promoter strength in conventional bicistronic vector systems (Ansari and Hampsey, 2005). However, few studies have examined the combined effects of promoter length and PAS context on the stoichiometric balance of dual cistron products. Our work addresses this research gap: promoter truncation alters upstream transcriptional magnitude, while functional PAS determines whether such transcriptional changes reshape relative downstream gene expression. The Tet-On reporter system utilized in this study provides a dynamic platform to interrogate transcription-polyadenylation coupling, complements static promoter deletion assays, and verifies consistent regulatory patterns across different promoter backbones (Xu et al., 2002). Despite the widespread application of bicistronic reporters to dissect gene regulatory networks, vector design studies have largely overlooked the critical role of PAS sequence in shaping inter-cistron expression ratios (Alexander et al., 2010).

Our findings appear inconsistent with earlier reports claiming IRES translation is unaffected by transcriptional changes (Rübsamen et al., 2012). This discrepancy arises from fundamental differences in plasmid design. The bicistronic vectors used in our study carry functional PAS that stabilize bicistronic mRNA and modulate cistron abundance ratios, whereas most previous studies likely relied on PAS-deficient vector backbones. Loss of polyadenylation signals triggers non-specific transcript degradation, which masks transcription-dependent shifts in IRES-derived protein yields (Zheng et al., 2018).

Several limitations of this study should be acknowledged. Our conclusions are derived from custom synthetic bicistronic reporters, which were applied to quantify PAS strength and alternative polyadenylation patterns (Ji et al., 2009). This experimental strategy was selected because the coupled transcription-polyadenylation effect is difficult to isolate and analyze at endogenous genomic loci, and although these reporters showed that identified PAS elements are sufficient to mediate transcription-coupled polyadenylation regulation, they cannot fully recapitulate the complex regulatory microenvironment of native mRNA 3′regions. We also did not determine how promoter truncation affects RNA polymerase II (Pol II) recruitment, elongation rates, or cofactor assembly, and unresolved events such as promoter-proximal Pol II pausing require integrative Pol II ChIP-seq and nascent RNA-seq analyses for full mechanistic clarification (Park et al., 2004). Our findings are further restricted to the FSK-induced BeWo cells model, the most widely used *in vitro* system for trophoblast syncytialization, selected for its reproducibility, robust cell fusion phenotype, and extensive validation in prior studies. BeWo is nonetheless a choriocarcinoma line in which FSK signals primarily through the cAMP/PKA pathway, so the results should be read as evidence of a mechanism operating in this well-defined model rather than a direct reflection of placental development *in vivo*. Only nine trophoblast-associated genes were examined in this study. These genes were selected based on their documented roles in trophoblast function or syncytialization and for identifiable PAS elements in their 3′UTRs. Such a candidate-gene approach cannot reveal the genome-wide scope of PAS-dependent remodeling and is potentially subject to selection bias. Finally, although the present work defines core crosstalk between promoter activity, PAS sequence identity, and bicistronic expression, the molecular cascade by which a PAS transmits promoter signals and balances bicistronic output remains uncharacterized. We did not profile differential binding between PAS variants and polyadenylation complexes under variable transcription loads, nor identify the regulatory factors governing PAS transcriptional sensitivity. These mechanistic questions can be resolved via co-immunoprecipitation and single-molecule fluorescence imaging assays (Schönemann et al., 2014; Chan et al., 2014). Additionally, the interplay between IRES-mediated translation and polyadenylation remains unexplored, including PAS-dependent modulation of mRNA stability, poly(A) tail length-mediated IRES structural remodeling, and ITAF recruitment—processes that can be clarified through polysome profiling and RIP-seq analyses (Bradrick et al., 2007). To address these limitations, future studies will validate our key findings in primary human cytotrophoblasts and trophoblast stem cells, induce syncytialization through EGF or density-dependent spontaneous fusion, and expand the differentiation marker panel to include *GCM1*, *Syncytin-1 (ERVW-1), MFSD2A, hCGα (CGA)*, and *CSH1*. Genome-wide PolyA-seq before and after syncytialization will map the broader landscape of alternative polyadenylation, while perturbation of the polyadenylation machinery (CPSF subunits, CSTF2, NUDT21/CFIm25) and CRISPR-based editing of endogenous PAS elements (e.g., in *GCM1* or *CGB*), followed by syncytialization and quantification of β-hCG secretion, syncytin-1/2 expression, and cell fusion efficiency will move beyond association toward causation. In the longer term, human placental organoids and *ex vivo* villous explants will allow PAS-dependent regulation to be examined in a three-dimensional, tissue-like context. Together, these follow-up experiments will establish the necessity of specific PAS elements in trophoblast differentiation, complementing the sufficiency evidence demonstrated by our reporter assays.

In summary, this study uncovers a previously uncharacterized regulatory mechanism that integrates transcriptional magnitude and PAS sequence identity to control bicistronic transcript stoichiometry. By combining static promoter truncation assays and dynamic Tet-On inducible system, we identified an uncoupled regulatory pattern between upstream transcription and IRES-dependent downstream translation, wherein functional PAS elements act as an indispensable, context-dependent bridge that converts promoter activity fluctuations into balanced cistron expression. Notably, our graded transcription assays identified a novel hierarchical PAS regulatory paradigm: canonical and suboptimal PAS motifs confer robust 3′-end processing tolerance against transcription elevation, whereas non-canonical PAS variants are transcription-sensitive and exhibit attenuated processing efficiency under enhanced transcription. More interestingly, during FSK-induced BeWo cell syncytialization, we found that endogenous pro-syncytialization genes preferentially utilize high-activity canonical PASs, which are selectively reinforced upon transcriptional activation to enrich pro-differentiation transcripts and guarantee orderly syncytiotrophoblast maturation. Collectively, these findings define a novel hierarchical transcription–polyadenylation regulatory axis, address critical knowledge gaps in understanding coordinated control of transcription and mRNA processing during cell fate specification, and lay mechanistic groundwork for placental developmental regulation and rational optimization of bicistronic vectors.

## Conclusion

This study identified an unrecognized regulatory circuit coupling transcription strength to PAS sequence identity to coordinate bicistronic transcript stoichiometry. Using promoter truncation and Tet-On induction systems, we confirmed that upstream transcription and IRES-dependent translation are decoupled, with PAS elements acting as a context-dependent bridge that converts promoter activity shifts into balanced cistron expression. Graded transcription assays revealed a tiered PAS processing model: canonical and suboptimal PASs maintain stable 3′-end processing at high transcription levels, whereas non-canonical PASs display compromised processing efficiency with rising transcription. During FSK-induced BeWo cell syncytialization, pro-syncytial genes are equipped with potent PASs that exhibit enhanced processing under elevated transcription, whereas anti-syncytial genes contain weak PASs showing little response to heightened transcriptional input. Therefore, our findings define a hierarchical transcription-polyadenylation axis that may be associated with the regulation of placental trophoblast syncytialization. These data address critical gaps in mRNA processing and lay the foundation for studying the transcriptional regulatory mechanisms underlying trophoblast differentiation.

## Data Availability

The original contributions presented in the study are included in the article/Supplementary Material, further inquiries can be directed to the corresponding authors.

## References

[B1] Alexander R. D. Barrass J. D. Dichtl B. Kos M. Obtulowicz T. Robert M. C. (2010). RiboSys, a high-resolution, quantitative approach to measure the in vivo kinetics of pre-mRNA splicing and 3'-end processing in Saccharomyces cerevisiae. RNA 16, 2570–2580. 10.1261/rna.2162610 20974745 PMC2995417

[B2] Ansari A. Hampsey M. (2005). A role for the CPF 3'-end processing machinery in RNAP II-dependent gene looping. Genes Dev. 19, 2969–2978. 10.1101/gad.1362305 16319194 PMC1315401

[B3] Beaudoing E. Freier S. Wyatt J. R. Claverie J. M. Gautheret D. (2000). Patterns of variant polyadenylation signal usage in human genes. Genome Res. 10, 1001–1010. 10.1101/gr.10.7.1001 10899149 PMC310884

[B4] Bradrick S. S. Dobrikova E. Y. Kaiser C. Shveygert M. Gromeier M. (2007). Poly(A)-binding protein is differentially required for translation mediated by viral internal ribosome entry sites. RNA 13, 1582–1593. 10.1261/rna.556107 17652408 PMC1950770

[B5] Chan S. L. Huppertz I. Yao C. Weng L. Moresco J. J. Yates J. R. (2014). CPSF30 and Wdr33 directly bind to AAUAAA in mammalian mRNA 3' processing. Genes Dev. 28, 2370–2380. 10.1101/gad.250993.114 25301780 PMC4215182

[B6] Cheng L. C. Zheng D. Baljinnyam E. Sun F. Ogami K. Yeung P. L. (2020). Widespread transcript shortening through alternative polyadenylation in secretory cell differentiation. Nat. Commun. 11, 3182. 10.1038/s41467-020-16959-2 32576858 PMC7311474

[B7] Costa M. A. (2016). The endocrine function of human placenta: an overview. Reprod. Biomed. Online 32, 14–28. 10.1016/j.rbmo.2015.10.005 26615903

[B8] Deng Z. Zhang S. Gu S. Ni X. Zeng W. Li X. (2018). Useful bicistronic reporter System for studying Poly(A) site-defining cis elements and regulation of alternative polyadenylation. Int. J. Mol. Sci. 19, 279. 10.3390/ijms19010279 29342112 PMC5796225

[B9] Di Giammartino D. C. Nishida K. Manley J. L. (2011). Mechanisms and consequences of alternative polyadenylation. Mol. Cell 43, 853–866. 10.1016/j.molcel.2011.08.017 21925375 PMC3194005

[B10] Dichtl B. Blank D. Sadowski M. Hübner W. Weiser S. Keller W. (2002). Yhh1p/Cft1p directly links poly(A) site recognition and RNA polymerase II transcription termination. EMBO J. 21, 4125–4135. 10.1093/emboj/cdf390 12145212 PMC126137

[B11] Greger I. H. Proudfoot N. J. (1998). Poly(A) signals control both transcriptional termination and initiation between the tandem GAL10 and GAL7 genes of Saccharomyces cerevisiae. EMBO J. 17, 4771–4779. 10.1093/emboj/17.16.4771 9707436 PMC1170806

[B12] Hoarau J. J. Cesari M. Caillens H. Cadet F. Pabion M. (2004). HLA DQA1 genes generate multiple transcripts by alternative splicing and polyadenylation of the 3' untranslated region. Tissue Antigens 63, 58–71. 10.1111/j.1399-0039.2004.00140.x 14651525

[B13] Huang Z. Moya C. Jayaraman A. Hahn J. (2010). Using the Tet-On system to develop a procedure for extracting transcription factor activation dynamics. Mol. Biosyst. 6, 1883–1889. 10.1039/c003229h 20552111

[B14] Jean D. Rousselet N. Frade R. (2008). Cathepsin L expression is up-regulated by hypoxia in human melanoma cells: role of its 5'-untranslated region. Biochem. J. 413, 125–134. 10.1042/BJ20071255 18366346

[B15] Ji Z. Lee J. Y. Pan Z. Jiang B. Tian B. (2009). Progressive lengthening of 3' untranslated regions of mRNAs by alternative polyadenylation during mouse embryonic development. Proc. Natl. Acad. Sci. U.S.A. 106 (17), 7028–7033. 10.1073/pnas.0900028106 19372383 PMC2669788

[B16] Ji Z. Luo W. Li W. Hoque M. Pan Z. Zhao Y. (2011). Transcriptional activity regulates alternative cleavage and polyadenylation. Mol. Syst. Biol. 7, 534. 10.1038/msb.2011.69 21952137 PMC3202805

[B17] Juge F. Zaessinger S. Temme C. Wahle E. Simonelig M. (2002). Control of poly(A) polymerase level is essential to cytoplasmic polyadenylation and early development in Drosophila. EMBO J. 21, 6603–6613. 10.1093/emboj/cdf633 12456666 PMC136937

[B18] Kazerouninia A. Ngo B. Martinson H. G. (2010). Poly(A) signal-dependent degradation of unprocessed nascent transcripts accompanies poly(A) signal-dependent transcriptional pausing in vitro. RNA 16, 197–210. 10.1261/rna.1622010 19926725 PMC2802029

[B19] Lainé J. P. Singh B. N. Krishnamurthy S. Hampsey M. (2009). A physiological role for gene loops in yeast. Genes Dev. 23, 2604–2609. 10.1101/gad.1823609 19933150 PMC2779762

[B20] Lamas-Maceiras M. Singh B. N. Hampsey M. Freire-Picos M. A. (2016). Promoter-Terminator gene loops affect alternative 3'-End processing in yeast. J. Biol. Chem. 291, 8960–8968. 10.1074/jbc.M115.687491 26929407 PMC4861464

[B21] Larochelle M. Hunyadkürti J. Bachand F. (2017). Polyadenylation site selection: linking transcription and RNA processing via a conserved carboxy-terminal domain (CTD)-interacting protein. Curr. Genet. 63, 195–199. 10.1007/s00294-016-0645-8 27582274

[B22] Lin J. Yu Z. Ye C. Hong L. Chu Y. Shen Y. (2021). Alternative polyadenylated mRNAs behave as asynchronous rhythmic transcription in Arabidopsis. RNA Biol. 18, 2594–2604. 10.1080/15476286.2021.1933732 34036876 PMC8632115

[B23] Miao X. Hu G. (2025). Poly(A) tail regulation in stem cells and early development. Cell Regen. 14, 48. 10.1186/s13619-025-00250-0 41288893 PMC12647427

[B24] Mitschka S. Mayr C. (2022). Context-specific regulation and function of mRNA alternative polyadenylation. Nat. Rev. Mol. Cell Biol. 23, 779–796. 10.1038/s41580-022-00507-5 35798852 PMC9261900

[B25] Nag A. Narsinh K. Kazerouninia A. Martinson H. G. (2006). The conserved AAUAAA hexamer of the poly(A) signal can act alone to trigger a stable decrease in RNA polymerase II transcription velocity. RNA 12, 1534–1544. 10.1261/rna.103206 16775304 PMC1524889

[B26] Nagaike T. Logan C. Hotta I. Rozenblatt-Rosen O. Meyerson M. Manley J. L. (2011). Transcriptional activators enhance polyadenylation of mRNA precursors. Mol. Cell. 41, 409–418. 10.1016/j.molcel.2011.01.022 21329879 PMC3060669

[B27] O'Sullivan J. M. Tan-Wong S. M. Morillon A. Lee B. Coles J. Mellor J. (2004). Gene loops juxtapose promoters and terminators in yeast. Nat. Genet. 36, 1014–1018. 10.1038/ng1411 15314641

[B28] Park N. J. Tsao D. C. Martinson H. G. (2004). The two steps of poly(A)-dependent termination, pausing and release, can be uncoupled by truncation of the RNA polymerase II carboxyl-terminal repeat domain. Mol. Cell Biol. 24, 4092–4103. 10.1128/mcb.24.10.4092-4103.2004 15121832 PMC400489

[B29] Pattillo R. A. Gey G. O. Delfs E. Mattingl R. F. (1968). Human hormone production in vitro. Science 159, 1467–1469. 10.1126/science.159.3822.1467 5753554

[B30] Proudfoot N. J. Furger A. Dye M. J. (2002). Integrating mRNA processing with transcription. Cell 108, 501–512. 10.1016/s0092-8674(02)00617-7 11909521

[B31] Rehfeld A. Plass M. Krogh A. Friis-Hansen L. (2013). Alterations in polyadenylation and its implications for endocrine disease. Front. Endocrinol. 4, 53. 10.3389/fendo.2013.00053 23658553 PMC3647115

[B32] Rübsamen D. Blees J. S. Schulz K. Döring C. Hansmann M. L. Heide H. (2012). IRES-dependent translation of egr2 is induced under inflammatory conditions. RNA 18, 1910–1920. 10.1261/rna.033019.112 22915601 PMC3446713

[B33] Sandberg R. Neilson J. Sarma A. Sharp P. A. Burge C. B. (2008). Proliferating cells express mRNAs with shortened 3' untranslated regions and fewer microRNA target sites. Sci. 320 (5883), 1643–1647. 10.1126/science.1155390 18566288 PMC2587246

[B34] Schönemann L. Kühn U. Martin G. Schäfer P. Gruber A. R. Keller W. (2014). Reconstitution of CPSF active in polyadenylation: recognition of the polyadenylation signal by WDR33. Genes Dev. 28, 2381–2393. 10.1101/gad.250985.114 25301781 PMC4215183

[B35] Sun Y. Zhang Y. Hamilton K. Manley J. L. Shi Y. Walz T. (2017). Molecular basis for the recognition of the human AAUAAA polyadenylation signal. Proc. Natl. Acad. Sci. U. S. A. 115 (7), E1419–E1428. 10.1073/pnas.1718723115 29208711 PMC5816196

[B36] Tan-Wong S. M. Zaugg J. B. Camblong J. Xu Z. Zhang D. W. Mischo H. E. (2012). Gene loops enhance transcriptional directionality. Science 338, 671–675. 10.1126/science.1224350 23019609 PMC3563069

[B37] Tang P. Zhou Y. (2022). Alternative polyadenylation regulation: insights from sequential polyadenylation. Transcription 13, 89–95. 10.1080/21541264.2022.2114776 36004392 PMC9715272

[B38] Tian B. Manley J. L. (2017). Alternative polyadenylation of mRNA precursors. Nat. Rev. Mol. Cell Biol. 18, 18–30. 10.1038/nrm.2016.116 27677860 PMC5483950

[B39] Wang R. Zheng D. Yehia G. Tian B. (2018). A compendium of conserved cleavage and polyadenylation events in mammalian genes. Genome Res. 28, 1427–1441. 10.1101/gr.237826.118 30143597 PMC6169888

[B40] Xu Z. L. Mizuguchi H. Ishii-Watabe A. Uchida E. Mayumi T. Hayakawa T. (2002). Strength evaluation of transcriptional regulatory elements for transgene expression by adenovirus vector. J. Control. Release. 81, 155–163. 10.1016/s0168-3659(02)00059-7 11992688

[B41] Yao C. Biesinger J. Wan J. Weng L. Xing Y. Xie X. (2012). Transcriptome-wide analyses of CstF64-RNA interactions in global regulation of mRNA alternative polyadenylation. Proc. Natl. Acad. Sci. U. S. A. 109, 18773–18778. 10.1073/pnas.1211101109 23112178 PMC3503179

[B42] Zheng D. Wang R. Ding Q. Wang T. Xie B. Wei L. (2018). Cellular stress alters 3'UTR landscape through alternative polyadenylation and isoform-specific degradation. Nat. Commun. 9, 2268. 10.1038/s41467-018-04730-7 29891946 PMC5995920

