# Supplementary material for: Transcription-dependent polyadenylation signal activity contributes to transcriptomic regulation during Forskolin-induced BeWo cell syncytialization
Source: Front Cell Dev Biol. 2026 Sep 25;14:1958080. doi: 10.3389/fcell.2026.1958080 (PMC13650466; doi:10.3389/fcell.2026.1958080)
Supplement: Supplementary file 1 [file Supplementaryfile1.docx]

**Transcription-dependent polyadenylation signal activity contributes to transcriptomic regulation during Forskolin-induced BeWo cell syncytialization**

Juan Liu ^1^, Guolin Ye ^1,2^, Xin Liu ^3,4^, Haiming Jing ^1^^,2^, Shan Gao ^1,2^, Zhongyuan Deng ^3,*^, Junyu Ning ^1,2,*^

^1^ Beijing Key Laboratory of Diagnostic and Traceability Technologies for Food Poisoning, Beijing Center for Disease Prevention and Control, Beijing 100013, China;

^2^ School of Public Health, Capital Medical University, Beijing 100069, China;

^3^ School of Agriculture and Biomanufacturing, Zhengzhou University, Zhengzhou 450001, China;

^4^ Biology and Agriculture Research Center, University of Science and Technology Beijing, Beijing, China;

^*^ Correspondence: dzy@zzu.edu.cn (Z.D.); njy_med@hotmail.com (J.N.)

**Supplementary Table A1.** The primer used in this article.

| **SV40 promoter gradient deletion primer** | | |
| --- | --- | --- |
| **Gene name** | | **Primer sequence** |
| delta100-Forward primer | | AGAGTGCACCATATGCAGA--AGTATGCAAA-GCATGCATCTC |
| delta200-Forward primer | | AGAGTGCACCATATGTCTCCGCCCC-ATGGCTGACTAAT |
| CMV- Reverse primer | | TTCACACCGCATATGATTCCACAGC-TGGTTCTTTCCGC |
| CMV- identification- Forward primer | | GCCTGAAATAACCTCTGAAAGAG |
| CMV- identification- Reverse primer | | TACCTGCCCAGTGCCTCACGAC |
| **Tet-On inducible dual-luciferase vectors construct** | | |
| **Gene name** | **Primer sequence** | |
| check2-reverse direction-Forward primer | ACCATGGCCGATGCTAAGAACATTA | |
| check2-reverse direction-Reverse primer | AGATCCTCACACAAAAAACCAACACAC | |
| TET-bglII-Forward primer | GCGCGACAGATCTCAGAGATCCAGTTTATCGATAA | |
| TET-nheI-Reverse primer | GCGATAGCTAGCGAATTCTGTGGCAAGCTTATCATCGTGTTTTTCAAAGG | |
| psi-tet-CMVdel-Forward primer | GAATTCGCTAGCTATCGCACCATGGCTTCCAAGGTGTACGACCCCGAG | |
| psi-tet-CMVdel-Reverse primer | CTGAGATCTGTCGCGCGTCGAGCCATGTGAGCAAAAGGCCAGCAAA | |
| IRES-homology-Forward primer | GTTTTTTGTGTGAGATCTGAATTCGCCC-  CTCTCCCTCCCCCCCCCCTAA | |
| IRES- homology- Reverse primer | CTTAGCATCGGCCATGGTTGTGGCCATATTATCATCGTGTTTTTC | |
| **PAS variant reporter plasmids construct** | | |
| **Gene name** | **Primer sequence** | |
| psi-wide-Forward primer | CTCCGCTCGAGAATAAAATATCTTT  ATTTTCATTACATCTGTGTGTTGG | |
| psi-Reverse primer | CTGCGGAATTCTCACACAAAAAACCAACACACAGATGTAATGAAAAT | |
| psi-rad-Forward primer | CTCCGCTCGAGNNNNNNATATCTT-TATTTTCATTACATCTGTGTGTTGG | |
| psi-del-Forward primer | CTCCGCTCGAGATATCTTTATTTTCA-TTA-CATCTGTGTGTTGG | |
| psi-mut1-Forward primer | CTCCGCTCGAGANTAAAATATCT-TTATTTTCATTACATCTGTGTGTTGG | |
| psi-mut3-Forward primer | CTCCGCTCGAGNANAANATAT-CTTTATTTTCATTACATCTGTGTGTTGG | |
| IRES-Reverse primer | GACGGCAATATGGTGGAAAA | |
| **Primers for amplification of upstream and downstream transcript fragments of target genes** | | |
| **Gene name** | **Primer sequence** | |
| GCM1-Forward primer-1 | GCACAGGGGCTTCTGTTTTATCC | |
| GCM1-Reverse primer-1 | AAACATGGCCGGCCAATCATTG | |
| GCM1-Forward primer-2 | GTCTCCTAGTTCTAGTCTCCCCT | |
| GCM1-Reverse primer-2 | GCAGAGAACATTTCTAATATC | |
| CGA-Forward primer-1 | TGCAGCTTTACTGCCTTCCTCC | |
| CGA-Reverse primer-1 | GCAGTGGAACAAGCTAAATGCTG | |
| CGA-Forward primer-2 | GTGTTTGGTGACCTGACATTGG | |
| CGA-Reverse primer-2 | GTCTTATAAGTAGCCCACTGGC | |
| CGB-Forward primer-1 | TCAGCTGTCAATGTGCACTCTGC | |
| CGB-Reverse primer-1 | CTTTGAGGAAGAGGAGTCCTGG | |
| CGB-Forward primer-2 | GCAACCACACACACACAGGATGGG | |
| CGB-Reverse primer-2 | ATCAGCCATCGCACCTGGCCAT | |
| ERVW-1-Forward primer-1 | GGCTGACTAAGAATCCCTAAGCC | |
| ERVW-1-Reverse primer-1 | GATTGGTCAGGTGTGAGCTAAG | |
| ERVW-1-Forward primer-2 | GCTAAAGGCTTGCCATTGTTCC | |
| ERVW-1-Reverse primer-2 | TTCAAGGAATGGAACCTTGGGCC | |
| ERVFRD-1-Forward primer-1 | TGCCTTGGACTATCATGCTACC | |
| ERVFRD-1-Reverse primer-1 | AATCCAATGGGTCTGCAGCAGAC | |
| ERVFRD-1-Forward primer-2 | CCACCTGTACTGAAGCCATCCTC | |
| ERVFRD-1-Reverse primer-2 | GGAACCATGGAGATGTAAATGGAC | |
| CSH1-Forward primer-1 | GGAAGGCATCCAAACGCTGATGG | |
| CSH1-Reverse primer-1 | CAGTAGAGCAGCCCGTAGTTCTT | |
| CSH1-Forward primer-2 | AGGCCATTCCACCAGCTCAGTGG | |
| CSH1-Reverse primer-2 | TGCCTCTGCACTTAATAGCCCAT | |
| CDH1-Forward primer-1 | CGGCAGTTCAAGCTATATCGA | |
| CDH1-Reverse primer-1 | CACAATTATCAGCACCCACACATG | |
| CDH1-Forward primer-2 | GCCTGTAATCTCAGCACTTCGGG | |
| CDH1-Reverse primer-2 | TTGTGGAGACAGGGTTTCACT | |
| TP63-Forward primer-1 | CCTCATGGCAGCCTACTCTCCTTG | |
| TP63-Reverse primer-1 | CAGCTAACTGCATTGAGCTGAG | |
| TP63-Forward primer-2 | GGAAAGACCAGAATGGCATTTTC | |
| TP63-Reverse primer-2 | GAGGTCACTGCTGAGTATCATCGG | |
| TEAD4-Forward primer-1 | GCCTATGTCTTTGAGGTGTCAG | |
| TEAD4-Reverse primer-1 | CGTTTCCTGCACACACGTCTCTTC | |
| TEAD4-Forward primer-2 | CAGGACGGTTCACACAGAGAGG | |
| TEAD4-Reverse primer-2 | GAGATGGACGGAAACTATCCAGAG | |
